# Semi-supervised incremental learning with few examples for discovering medical association rules

**DOI:** 10.1186/s12911-022-01755-3

**Published:** 2022-01-24

**Authors:** Ricardo Sánchez-de-Madariaga, Juan Martinez-Romo, José Miguel Cantero Escribano, Lourdes Araujo

**Affiliations:** 1 Telemedicine and e-Health Research Unit, Monforte de Lemos 5, Instituto de Salud Carlos III, 28029 Madrid, Spain; 2 Natural Language Processings and Information Retrieval Group, Universidad Nacional de Educación a Distancia, 28040 Madrid, Spain; 3 Preventive Medicine Service, Hospital Universitario La Paz-Carlos III-Cantoblanco, 28046 Madrid, Spain; 4 Instituto Mixto UNED-ISCIII, IMIENS, 28029 Madrid, Spain

**Keywords:** Medical records, Association rules discovery, Machine learning, Semi-supervised approach

## Abstract

**Background:**

Association Rules are one of the main ways to represent structural patterns underlying raw data. They represent dependencies between sets of observations contained in the data. The associations established by these rules are very useful in the medical domain, for example in the predictive health field. Classic algorithms for association rule mining give rise to huge amounts of possible rules that should be filtered in order to select those most likely to be true. Most of the proposed techniques for these tasks are unsupervised. However, the accuracy provided by unsupervised systems is limited. Conversely, resorting to annotated data for training supervised systems is expensive and time-consuming. The purpose of this research is to design a new semi-supervised algorithm that performs like supervised algorithms but uses an affordable amount of training data.

**Methods:**

In this work we propose a new semi-supervised data mining model that combines unsupervised techniques (Fisher’s exact test) with limited supervision. Starting with a small seed of annotated data, the model improves results (F-measure) obtained, using a fully supervised system (standard supervised ML algorithms). The idea is based on utilising the agreement between the predictions of the supervised system and those of the unsupervised techniques in a series of iterative steps.

**Results:**

The new semi-supervised ML algorithm improves the results of supervised algorithms computed using the F-measure in the task of mining medical association rules, but training with an affordable amount of manually annotated data.

**Conclusions:**

Using a small amount of annotated data (which is easily achievable) leads to results similar to those of a supervised system. The proposal may be an important step for the practical development of techniques for mining association rules and generating new valuable scientific medical knowledge.

**Supplementary Information:**

The online version contains supplementary material available at 10.1186/s12911-022-01755-3.

## Background

Discovering the set of patterns or regularities that underlie raw data is the aim of Data Mining. One of the main ways to represent structural patterns underlying raw data is by Association Rules, which express dependencies or correlations between facts or observations in the data. Such dependency analysis is central to empirical science. Medical professionals want to identify factors or diseases that predispose to or prevent other diseases, and genetic researchers are interested in which gene groups correlate. For example, in the medical field, we can find an AR:

$$\begin{aligned} arthralgia, female gender, achneform eruptions \rightarrow PCDF \end{aligned}$$

which asserts that there is a positive dependency between high levels of polychlorinated dibenzofuran (PCDF) and the presence of arthralgia and acne eruptions in female patients. This was demonstrated in the oil poisoning environmental case that happened in Japan during the sixties [1]. Even if there are various reasons why such a dependency relationship exists between different symptoms, the very existence of the relationship provides valuable information. It can influence decisions on medical diagnosis or treatments [2]. ARs, comprised of a few elements with some relationship between them, are much easier to interpret than other methods for identifying correlations, such as those based on automatic learning (Bayesian Networks, Support Vector Machines or Neural Networks). For instance, a database of such ARs could be set the following query: “find all rules that have problem pharyngitis as consequent”, and these rules could identify which medical symptoms or problems should be treated or determined in order to prevent or to diagnose pharyngitis.

There are several algorithms based on heuristics statistical models [3] that provide the complete set of ARs compatible with a database of groups of elements (events, medical conditions, features, etc.) that have occurred at the same time. However, many of these rules are irrelevant and may have happened by chance. A solution to this problem could be to train a Machine Learning system (ML) to identify relevant rules. However, this would require training data from which to learn. Due to the large amount of ARs generated from a database of coinciding elements, such rules are rarely relevant or negligible, since it is a costly and time-consuming process for medical experts.

The objective of this work is to design a new semi-supervised iterative ML algorithm, i.e. an algorithm that minimizes the amount of tagged ARs to be supplied as input. It only needs a tiny initial seed of tagged ARs that self-trains the algorithm in an incremental and iterative way. This is called bootstrapping [4], and it means that the economic and time costs of discovering new valid ARs would diminish drastically, and it could make the task more practicable.

The proposed algorithm is based on a combination of supervised and unsupervised techniques which can detect the most reliable information which is then used to improve the incremental training of the system. The supervised system is based on a number of relevant AR features. We have evaluated the system using real data from different sections of a hospital, and such data being homogenized, anonymized and standardized into EHR extracts. The data refers to real problems of hospital patients. We performed an exhaustive evaluation of the proposal, comparing the results of an unsupervised approach (0.63 F-measure), with a fully supervised one (0.71 F-measure) and also with the proposed semi-supervised system (0.75 F-measure).

The new semi-supervised algorithm performs in a similar way to fully supervised ML algorithms on the same corpus, but uses a much smaller amount of manually tagged ARs, thus making the discovery of new medical knowledge easier to achieve.

The formal definitions of ARs and the concept of goodness measure, related to an AR can be found in Additional file 1: Supplementary Material, section 1. Different goodness measures are available, the most widely used are the ${\chi }^2$-measure [5], for high absolute frequencies, and Fisher’s exact test [6] when these frequencies are low in general.

Given a set of data, several algorithms may be used to generate ARs implied by the data. However, a brute force search algorithm may generate such a high number of ARs that the problem is often called the *curse of dimensionality*. Some algorithms, such as *FP-growth* [7], use a number techniques to limit the number of rules produced. These include a minimum frequency threshold, also called *support* of the rule; or a minimum *confidence* of the rule.

However, none of these two requirements guarantees the existence of a positive dependence between the antecedent and the consequent of the rule, and indeed the rule might have been generated by chance. Even after selecting those rules included in the goodness measure there may be two kinds of errors. Type 1 errors (false positives) refer to rules that pass the validation test but are false, and type 2 errors (false negatives) refer to invalidated but true rules [8]. These two types of errors are usually complementary. Accordingly, the discovered ARs should always be pruned in a post-processing phase using a statistical test (goodness measure) such as the ${\chi }^2$ test or Fisher’s exact test.

### Selection of significant patterns

In order to alleviate the false-positive problem in the discovery of association rules several testing correction techniques have been proposed [9]. Most of them are based on the use of *p* values. The *p* value of an association rule R is the probability of observing R, or one rule which is stricter than R, when the two sides of R are independent. A low *p* value rule is unlikely to occur if its two sides are independent. Accordingly, since the rule has been found in the data, it is unlikely that its two sides are independent, and the association is likely to be true. By way of contrast, a high *p* value does not provide information about the independence of the two sides of the rule, and such rules can be discarded. A commonly used *p* value threshold [10] is 0.05. Some of the most frequently used statistical tests for computing *p* values are Pearson chi-square test of independence [5, 11] and Fisher’s exact test [6]. These tests compute the *p* value from the discrepancies between observed and expected values. Whereas chi-square is an approximation for large sample sizes, Fisher’s exact test, provides an exact *p* value for any sample size.

A technique for reducing the number of false positives proposed by Webb [12], is based on separating the available data into exploratory and holdout sets. The exploratory set is used to discover rules using standard algorithms for association rules, such as FPGrowth [7]. The holdout set is then used to compute the statistical significance of the discovered rules using a standard test. Finally, by setting an appropriate threshold for the required statistical significance, the most promising rules are selected.

Fisher’s test provides the significance of the association (contingency) between the two ways of classifying data. The computation of the test is usually based on the contingency table which records the different classes. The *p* value is computed as the hypergeometric distribution of the numbers contained in the cells of the table.

### Semi-supervised learning

Standard supervised ML algorithms trying to discover new good (true) rules (i.e. new medical knowledge) have a severe problem namely the excessive amount of necessary training. The amount of data used to train a model has a direct impact on its performance. Supervised systems trained on large amounts of annotated data outperform unsupervised systems, as they rely on more information related to the problem in question. However, human-annotated data is expensive and often difficult to obtain. This is because of the inherent complexity of knowledge-codifying rules and also the very high number of them being produced. Semi-supervised learning techniques can be an alternative when only limited amounts of annotated data are available. These techniques enhance a small amount of annotated data with a large amount of unlabeled data [4, 13]. This idea is related to other forms of semi-supervised learning, such as co-learning and mutual bootstrapping. The co-training approach [14] looks at multiple representations of the same data. During the co-training process, two classifiers are trained on the same data using different feature sets. These two classifiers then bootstrap each other and make predictions on unseen examples thereby feeding each other. Data labeled with high confidence by one classifier is given the other as training data. Another approach is mutual bootstrapping [15] which aims to learn different types of knowledge simultaneously by alternatively leveraging one type of knowledge to learn the other. Our proposal differs from these other approaches, since we do not combine two classifiers, but a supervised method with a non-supervised one. However, these provide different types of knowledge and are also applied alternatively (in a series of iterations) as they are in the mutual bootstrapping approach.

### Algorithms for association rule mining

Association rule mining (ARM) is one of the most popular methods used to extract knowledge from large databases [3] . In 1993 Agrawal et al. proposed the Apriori algorithm to extract frequent rules and patterns from databases [16]. Many researchers have tried to improve this process, including trying to generate ARs using faster algorithms such as FPGrowth or reducing the large number of rules generated [7, 17–23].

Examples of practical use of standard AR mining in the medical field include the identification of clinically accurate association between medications, laboratory results and diseases [24, 25] and clinical findings and chronic diseases [26]. Networks of such disease relationships are also visualized [27]. AR generation algorithms such as A-priori [16] or FPGrowth [7] have also been used to establish relationships between healthcare parameters and specific problems, such as heart disease [28], brain tumours [29], HIV [30], oral cancer [31], type 2 diabetes [32] or Alzheimer’s disease [33]. The difficulty of controlling the proliferation of type 1 errors (false positives) is closely related to the subject of this paper and is addressed in [34] with non-definitive results (i.e. this is an active research topic). In [35] it is applied to the specific problem of mining a medical image dataset. Guo et al. [36] address the relationship between readmission and other features in diabetics’ patient data, reducing the readmission of such patients. In [37] the best AR mining algorithm is tested and chosen using a number of different criteria.

## Methods

**Fig. 1:**
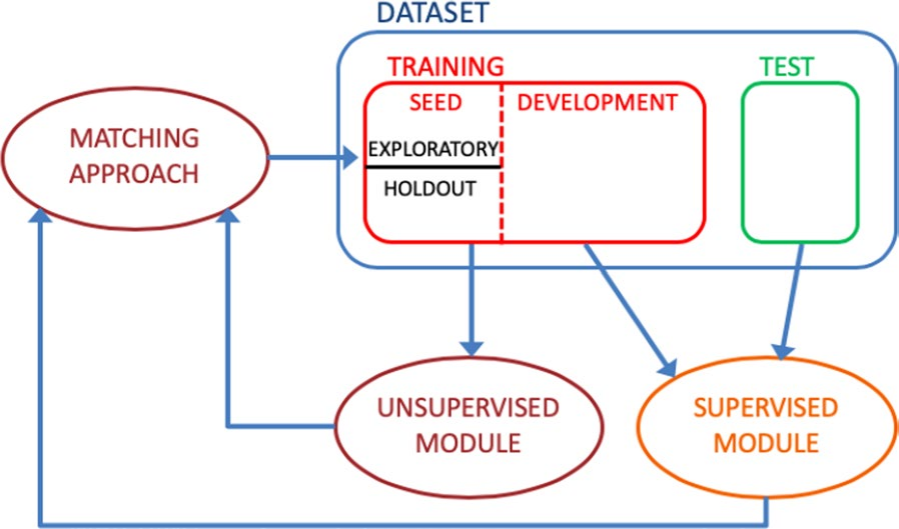
Interaction between the dataset and the unsupervised and supervised modules

The EXTRAE algorithm presented in this paper is a semi-supervised system comprised of two modules: one that implements an unsupervised method and another that implements a supervised method. First we will see first the unsupervised module, then the supervised module and finally the global system that we have called EXTRAE algorithm. Figure 1 shows a flow diagram with interaction between the dataset and the unsupervised and supervised modules.

Although use of the dataset is explained in detail in the following sections, a brief description of its use by the different modules of the system is included below. The dataset is initially divided into training (80%) and test (20%). As usual, the test set will be used to evaluate the performance of the system. The training set is in turn divided into seed and development, which will be used by the supervised module. The unsupervised module will only use the seed set. This seed set will be divided in equal parts by the unsupervised system as described in the following section. Seed and development sets are of variable size depending on the output of the matching approach, that combines the output of the unsupervised and supervised modules.

### Unsupervised module

In this work we have implemented an unsupervised module (Fisher’s exact test) in order to calculate the *p* value on a set of rules. Specifically, we have decided to use this *p* value to rank a set of association rules. We rank in ascending order and establish a threshold, and we decide to consider the *n* rules above that threshold (lower value) as true, and the *n* rules below that threshold (higher value) as false.

Specifically, we carried out an initial study of the results that we could obtain where there was no annotated data available and accordingly, we had to resort to unsupervised methods.

We apply the holdout technique proposed by Webb [12], splitting the dataset into exploratory and holdout parts, and applying the *p* value threshold on the holdout set in order to filter the rules extracted from the exploratory set.

Specifically, the following steps are performed:

- The dataset is divided in exploratory (50%) and holdout (50%).
- The FP-Growth algorithm is applied to extract the association rules in both sets. The use of this algorithm is that available in the SPMF software^1^. FP-Growth is an efficient algorithm for calculating frequently co-occurring items in a dataset.
- These two sets of rules allow us to apply the Fisher test to obtain the *p* values for the rules in the holdout set. Details about the computation of the test can be found in Additional file 1: Supplementary Material, section 2.
- Finally, the rules are sorted in the holdout set according to their *p* value. Then, a threshold for the *p* value is set in order to select the rules with higher significance in the holdout set, assuming that those selected rules are true and the rest are false. Here, the tricky point is the selection of an appropriate threshold.

**Fig. 2:**
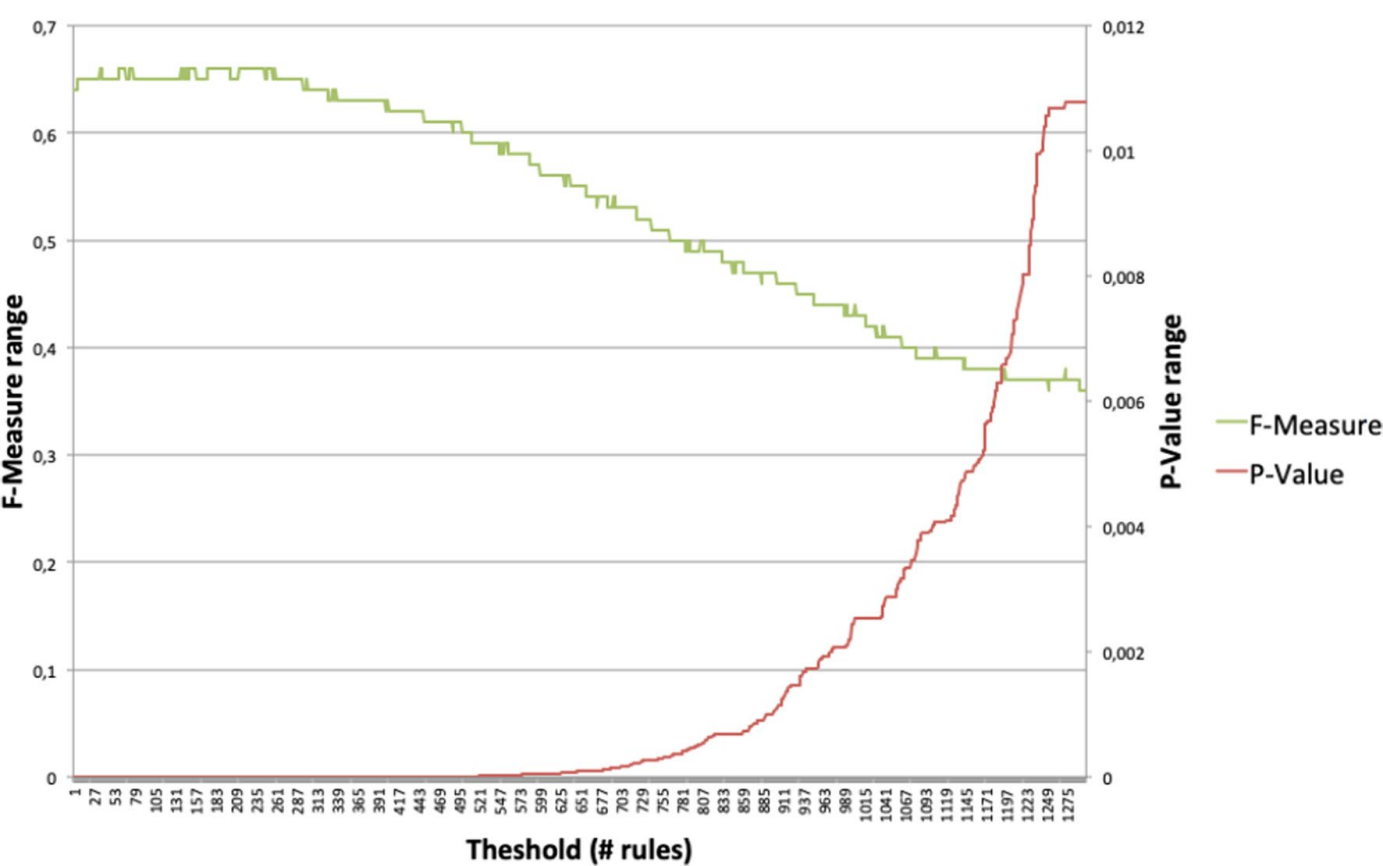
Evolution of the *p* value and the performance (F-measure) of the system depending on the threshold used

In order to illustrate the performance of the *p* value and its effect on system performance, an experiment with a sample labeled dataset is carried out. The *threshold* indicates the split between rules considered true and false. A rule with a *p* value lower than the *threshold* is considered as true, whereas a rule with a *p* value higher than the threshold is considered as false. Figure 2 shows the evolution of the *p* value and the performance (F-measure) of the system depending on the *threshold* used. It is clear from the optimal threshold value that a higher threshold has a negative impact on the performance of the system by reducing its f-measure. According to this experiment, the best *threshold* is 232 rules and this *threshold* corresponds to a *p* value of 1.42E-9. This setting achieves an evaluation score of 0.66.

### Supervised module

We have data which is annotated by doctors with true and false labels, and therefore we can implement a supervised approach. The objective of the EXTRAE algorithm is to start from a small set of manually annotated rules to increase their size in an unsupervised way and thus have a large set of rules automatically annotated as true or false. This supervised module (as we will see later) has two functions. On the one hand, it is used on the training set, along with another method, to predict the rules that can be added reliably to the seed set. It is also used on the whole test set for evaluation purposes by comparing the set of rules automatically annotated, with those annotated by a doctor.

We apply a Random Forest algorithm (see “Results” section), using the following set of features obtained from the fp-growth algorithm:

- Support. The support of an association rule “A and B $\rightarrow$ C” is the support of the set S = { A, B, C }. So the support of the rule is the (absolute or relative) number of cases in which the rule is correct (i.e. in which the presence of item C follows from the presence of items A and B).
- Confidence. The confidence of an association rule R = “X $\rightarrow$ Y” (with item sets X and Y) is the support of the set of all items that appear in the rule (the support of S = X $\cup$ Y) divided by the support of the antecedent (also called “if-part” or “body”) of the rule (here X).
- Lift. The lift value is the quotient of the posterior and the prior confidence of an association rule. That is, if “$\emptyset \rightarrow$ flu” has a confidence of 60% and “cough $\rightarrow$ flu” has a confidence of 72%, then the lift value (of the second rule) is 72/60 = 1.2.
- Number of antecedents. The number of antecedents of an association rule “A and B $\rightarrow$ C” is the number of elements of the set S ={ A, B }.
- Number of consequents. The number of consequents of an association rule “A and B $\rightarrow$ C” is the number of elements of the set S ={ C }.

### Semi-supervised approach

**Fig. 3:**
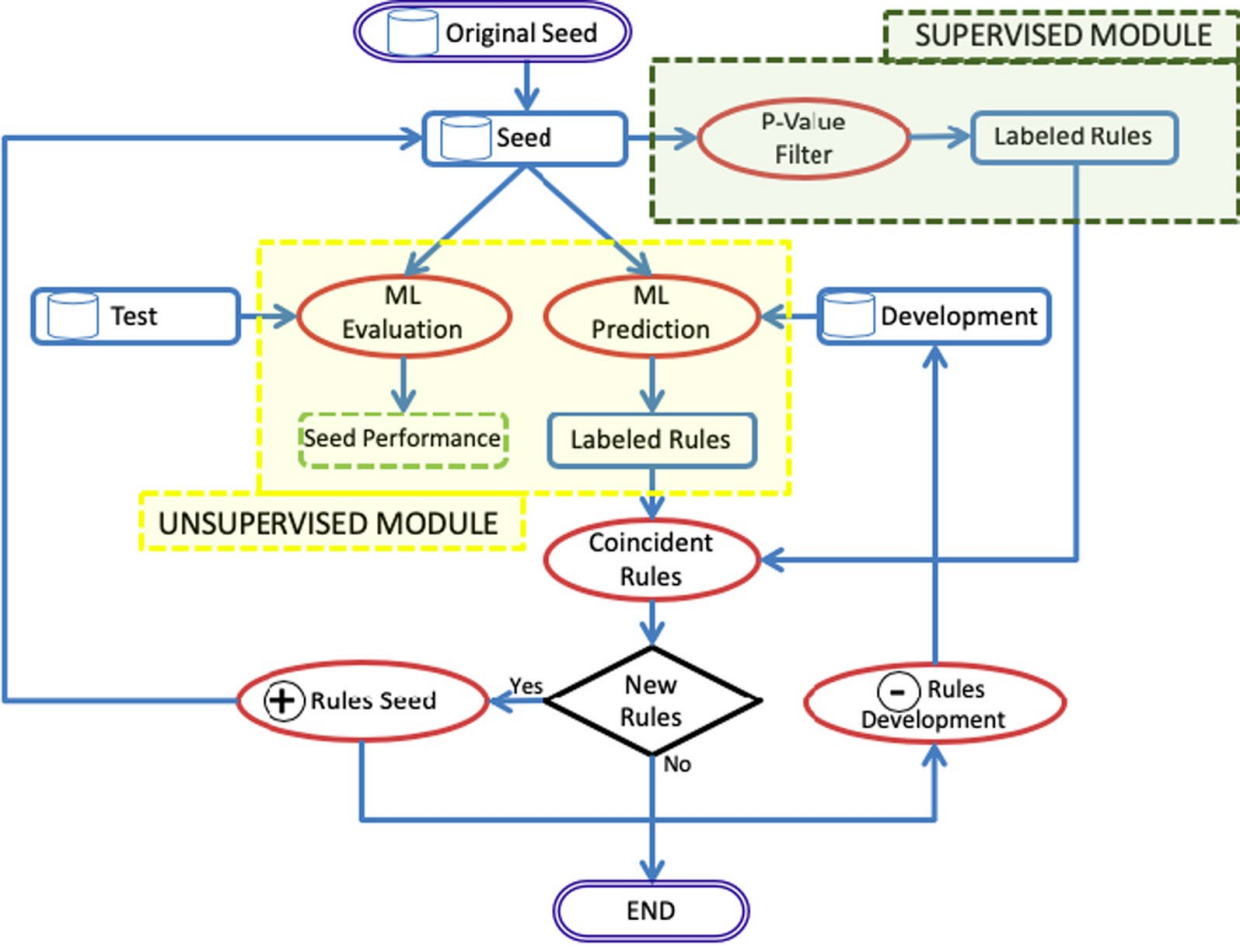
Flow diagram of incremental learning. Rounded rectangles show the beginning and the end of the iterations, rectangles are the rule sets, the broken line rectangle represents the seed set performance, ovals are processes, and the diamond represents a condition

Since manually classifying ARs as true or false by a health professional is an expensive and time-consuming task, we have resorted to a new semi-supervised approach that reduces the amount of annotated data needed. The idea is to use a small set of annotated rules to train a classifier and combine its predictions with those obtained using the *p* value method. Our hypothesis is that the cases in which both predictions coincide have a greater reliability and provide a new set of rules that can be used in turn to train the system. Figure 3 shows a flow diagram of the semi-supervised incremental learning approach.

Specifically, our semi-supervised approach involves the following steps:

- First, we have divided the annotated corpus (by doctors) into two different sets: training and test. Training set is 80% of the corpus while the test set is 20%. The training set is in turn divided into two sets: seed and development.
- We randomly select a small set of S rules from the training set, which is used as seed. This seed set is used to train the supervised module resulting in an ML model. The results will obviously be lower than those provided by a system trained with a larger set.
- The ML model (i.e. the machine learning system developed from seed rules) is then applied to predict the class (i.e. the True or False assignment) for each rule in the development set.
- First, the *p* value threshold is calculated based on the rules of the last computed seed set. This *p* value threshold is used for selecting the rules that are considered to be true or false. After sorting S according to their *p* value, we choose the *p* value as the threshold that maximizes the hits for the seed set (i.e. it divides the set into true and false rules with as many good predictions as possible).
- After that, the unsupervised module applies the *p* value filter to the predictions from the development set, as follows:
  We then select the cases from the development set in which the predictions of the supervised module and those of the unsupervised module, based on the *p* value filter, match: both are true or both are false. These coinciding rules are then added to the seed set and removed from the development set.
- The new seed set (previous seed set and coincident rules from development set) is used to train the supervised module again.
- The described process is repeated until the coincident set of rules obtained from the development set is empty (i.e. the seed set cannot grow anymore).

Each model trained with the incremental seed set is evaluated with the test set in order to have a reference to the performance improvement.

### Experimental framework

#### Dataset

In order to implement and test our semi-supervised ML algorithm we have used a standardized medical data corpus from the Fuenlabrada University Hospital (HUF) in Madrid, Spain. This corpus was constructed in a previous research project [38]. Electronic Health Records (EHR) from the HUF corpus are written in Spanish and normalized using the ISO/EN 13606 standard [39]. This standard follows a so-called dual model [40] that separates two levels of abstraction: one level of information, called Reference Model (RM) [41] and one level of knowledge, using archetypes [42]. The EHRs in this corpus correspond to primary attention, several specialized attention services and the pharmacy department of the hospital. The EHR extract files of the HUF corpus are XML files corresponding to one patient. Each patient may in fact have several EHR extracts containing his or her medical information, and thus each XML file holds the medical problems suffered by the patient to whom it belongs. We have used information from each medical problem (i.e. the name of the problem) to represent one different feature in our AR knowledge representation. This means that our medical data input to the FP-Growth algorithm generating the ARs is comprised of rows representing each patient and columns representing the name of each medical problem. In our ARs representation of the form

$$\begin{aligned} A B C \rightarrow D \end{aligned}$$

the symbols $A \cdots D$ correspond to the names of medical problems of one patient.

We call HUF-AR dataset to our manual annotated AR dataset generated out of the initial HUF data described in “Dataset” section. This HUF-AR dataset is generated applying the FP-Growth algorithm to the HUF data. We have set the FP-Growth parameters of support and confidence to 10% and 70%, respectively. Next, 1300 rules were randomly selected to be annotated by a doctor as true or false. Manual annotation was relatively simple as most ARs are composed of common diseases, typical of primary care. In addition, certain but trivial ARs were nevertheless classified as true, since they should contribute to the good behavior of the algorithm even though their intrinsic value was low.

The description of the medical problems are written in natural language which gives them great variability when referring to the same medical condition. In order to reduce this variability we have performed a preprocessing of the data, which is described in Additional file 1: Supplementary Material, section 3.

## Results

In this section we present the experiments carried out on the HUF corpus as well as the results obtained. Since the EXTRAE algorithm is comprised of an unsupervised module and a supervised module, we consider that it would be interesting to evaluate the impact of each of the modules separately. That is, to evaluate the unsupervised module as if it were an independent system and to do the same with the supervised module. In both cases (unsupervised and supervised module) a test set of 20% has been used. In this way, the following sections will show the results of this evaluation by modules and then the overall performance of the EXTRAE algorithm will be shown.

### Evaluation of the unsupervised module as an independent system

As seen in the previous sections, the unsupervised module uses implementation of the FP-Growth algorithm. This algorithm is an efficient and scalable method for mining the complete set of frequent patterns by pattern fragment growth. The parameters used for this algorithm are: Min. Support of 0.01; Min. confidence of 0.7; Minimum lift of 1; Max. antecedent length of 4; Max. consequent length of 1.

Table 1 shows results for the unsupervised method. Several *p* value thresholds have been analysed in order to prove the influence of this parameter. In the case of the unsupervised module, the threshold of the *p* value is applied directly on the test set (no training set is used).

**Table 1:** F-measure using different thresholds for the *p* value and using a test set (20%) in order to evaluate

Unsupervised module	
*p* Value	F-Measure
5E-2	0.384
1E-2	0.396
1E-3	0.492
1E-4	0.526
1E-5	0.557
1E-6	0.611
1E-7	0.615
1E-8	0.615
1E-9	0.623
1E-10	0.626
1E-11	**0**.**630**
1E-12	0.623
1E-13	0.619
1E-14	0.611

Best results appear in boldface

As per Table 1, the best threshold for the unsupervised method is a *p* value of $1E-11$, obtaining an F-measure of 0.63. This is a value consistent with the *p* value results shown in Fig. 2 for the whole corpus. Note that in this case, as it is an unsupervised method, the training set has not been used for any calculations. However, all operations have been carried out on the test set (20%). For this reason the results are slightly lower in this case.

### Evaluation of the supervised module as an independent system

The supervised method uses the features described above in “Supervised Module" section and the training and test sets are used in the usual way in any machine learning system. Because the EXTRAE algorithm works with a small set of association rules manually labelled by a doctor, we have designed an experiment to prove that a supervised system obviously gets worse results when the training set is smaller. In this experiment it is not intend to prove this fact but instead to analyze the difference in system performance depending on the size of the training set used. Table 2 shows the results for the supervised method depending on the size of the training set used. The test set has the same size (20%) in all the cases.

**Table 2:** Results for the *Random Forest* algorithm using different training set sizes and using the same test set (20%) for all the cases, based on their F-Measure, *AUC-ROC*, and *AU-PRC*

Supervised module			
Train/test %	F-measure	AUC-ROC	AU-PRC
5/20	0.63	0.67	0.68
10/20	0.64	0.67	0.68
20/20	0.66	0.68	0.69
30/20	0.66	0.69	0.71
40/20	0.67	0.70	0.72
50/20	0.66	0.71	0.70
60/20	0.68	0.71	0.73
70/20	0.70	0.72	**0**.**74**
80/20	**0**.**71**	**0**.**73**	**0**.**74**

Best results appear in boldface

In view of the results obtained by the supervised module in Table 2 the best training size is 80% (obtaining an F-measure of 0.71). In the case of the supervised module there is a meaningful difference between the training sizes used, and the performance grows as they do. Finally, by comparing the results of the unsupervised module, as expected, the supervised module obtains better results (0.71 vs 0.63). However, if we compare the performance of the unsupervised module with the supervised module when using a training set of the same size (20%), the results of both are similar (0.66 vs 0.63).

One of the relevant aspects when using a supervised system is the selection of the classification algorithm. The following section presents an experiment to compare a set of classification algorithms representing each of the existing classification algorithm families.

#### Supervised classification algorithms

Table 3 shows the results of the classification process using all the features introduced in this work and using several classification algorithms included in the Weka data mining tool [43]. A large number of classification algorithms from different families have been analyzed. The evaluation was carried out using the division training/test (80–20%) that achieved the best performance in Table 2 corresponding to the supervised system. Results show that Random Forest is the algorithm with the best performance. Thus Random Forest [44] is used in the following experiments where the supervised module is employed.

**Table 3:** Results using different classification algorithms on a split of 80–20% for training and test, based on their F-Measure, *AUC-ROC*, and *AU-PRC*

Algorithm	F-Measure	AUC-ROC	AU-PRC
NaiveBayesMultinomial	0.59	0.66	0.68
SimpleLogistic	0.57	0.62	0.63
MultilayerPerceptron	0.65	0.66	0.66
Logistic	0.62	0.67	0.69
VotedPerceptron	0.61	0.60	0.60
SVM	0.63	0.60	0.59
IBK	0.66	0.63	0.61
AdaBoostM1	0.58	0.65	0.63
ClassificationViaRegression	0.62	0.67	0.69
PART	0.66	0.67	0.65
Bagging+REPTree	0.70	0.69	0.69
RandomForest	**0**.**71**	**0**.**73**	**0**.**74**
J48	0.68	0.69	0.67
EXTRA Tree	0.69	0.66	0.63

Best results appear in boldface

### EXTRAE algorithm: semi-supervised incremental learning method

Table 4 shows the results of the semi-supervised method based on Incremental Learning (EXTRAE Algorithm). *Seed size* is the original size of the training set from which the set is automatically increased. *Iterations* show the number of times that a new rule needs to be added to the seed set in order that a set is reached to which no new rule can be added. The *p* value is calculated from the seed set. The results show the performance of the system after *n* iterations.

**Table 4:** Results of EXTRAE Algorithm on HUF corpus using different seed sizes, based on their F-Measure, *AUC-ROC*, and *AU-PRC*

HUF corpus					
Seed size	Iterations	*p* Value	F-Measure	AUC-ROC	AU-PRC
5	3	4.79E-13	0.73	**0**.**80**	**0**.**81**
10	7	4.79E-13	**0**.**75**	**0**.**80**	**0**.**81**
15	8	3.67E-13	0.72	0.79	0.80
20	14	3.67E-13	0.73	**0**.**80**	**0**.**81**
25	8	5.34E-10	0.74	0.79	0.80
35	6	3.3E-6	0.73	0.78	0.80
50	13	3.3E-6	0.74	0.78	0.80
75	4	3.35E-9	0.72	0.79	**0**.**81**
100	5	3.35E-9	0.69	0.79	**0**.**81**
125	5	3.35E-9	0.74	0.79	0.80
150	5	3.67E-13	0.72	0.79	0.80
175	4	3.67E-13	0.72	0.79	0.80
200	6	3.67E-13	0.74	**0**.**80**	**0**.**81**

Iterations is the max number of iterations reached and *p* value is obtained automatically using the filter approach on the seed set. Best results appear in boldface

From the results shown in Table 4, the best seed size is 10. A *p* value threshold of 4.79E-13 is calculated on this seed size and after 7 iterations an f-measure of 0.75 is obtained. The best results achieved with the supervised module were 0.71. The potential of the semi-supervised method based on Incremental Learning is thereby demonstrated.

The improvement in the results of the incremental-learning-based approach (EXTRAE Algorithm) in regards to the supervised module is remarkable, taking into account that in both cases the same features are used to train. The improvement is due to having used a method based on incremental learning, because the use of a *p* value threshold allows the selection of better rules for learning and therefore this trained model obtains better results. This is very similar to what happens in the semi-supervised Yarowsky algorithm [4] where it is of vital importance that very good examples are learned from the beginning of the algorithm in order to bootstrap it correctly and then to obtain good performance results [45].

Table 5 shows the partial results of the EXTRAE Algorithm in each iteration. In the first iteration 793 new rules are added and an F-measure of 0.70 is obtained. From the fourth iteration, the number of matching rules is greatly reduced and in this way the performance increases slowly until it reaches an F-measure of 0.75. Accuracy shows a great evolution from the original seed. In only one iteration it increases its performance by 16%, which proves the high quality of the added rules. Finally, the algorithm obtains an accuracy of 79%, improving the original accuracy by 21%.

**Table 5:** Evolution of learning from a seed set with 10 rules, based on their F-Measure, *AUC-ROC*, *AU-PRC*, and Accuracy

Iteration	Coincident rules	F-Measure	AUC-ROC	AU-PRC	Accuracy (%)
0	–	0.55	0.61	0.61	58
1	793	0.70	0.76	0.78	74
2	159	0.71	0.78	0.80	76
3	19	0.74	0.78	**0**.**81**	77
4	3	0.73	**0**.**80**	**0**.**81**	77
5	3	0.71	0.79	0.80	77
6	2	0.72	0.79	**0**.**81**	78
7	4	**0**.**75**	**0**.**80**	**0**.**81**	**79**

Coincident rules are those from the development set that have the same prediction and label based on the *p* value filter. Best results appear in boldface

## Conclusions

We propose a new semi-supervised system, called EXTRAE Algorithm, that requires a minimum amount of annotated data to obtain reliable association rules. This algorithm is comprised of two modules: a unsupervised module and a supervised module. The output of both modules is combined in order to obtain the best performance.

The idea behind the system is to combine the information provided by a supervised module trained with very few data and the information provided by an unsupervised module. Selecting the predictions on which both models agree, we enlarge the training data for the next step of the algorithm. The process continues until no new rules are selected in an iterative process.

We provide comparisons between an unsupervised model, a fully supervised model and the semi-supervised model (EXTRAE Algorithm). We find that a small seed with a size of between 10 and 20 rules is enough to achieve best results. This is because the EXTRAE algorithm only adds the best association rules to the set of rules that the supervised model learns about in order to make its predictions. From the results obtained, it is proven that the EXTRAE algorithm obtains better results as its initial set (seed set) of association rules grows.

This work marks an important breakthrough in the development of systems for mining association rules, since an extremely small amount of annotated data, which is easily achievable, leads to results similar to those of a supervised system.

It will be possible in the near future to design fast and cost-effective experiments to obtain and validate new medical knowledge (codified in the form of association rules) from large standardized medical databases, thereby permitting the advance of scientific medicine in general and Personalized and Precision Medicine (PPM) in particular.

In the future we plan to extend the algorithms to work with other kinds of features extracted from standardized medical databases, such as initial and final dates of problems, their duration or their gravity. This can indeed be applied to any other relevant feature from the patient’s EHR. We also plan to include data from the exposome, such as drugs, contaminants or daily lifestyle habits. We will perform the experiments on bigger and more specific databases, referring to a cohort especially selected to address a specific medical knowledge domain. We also plan to generate embeddings from medical reports. We will then explore any similarity between those embeddings according to the antecedents and consequents from the association rules as an alternative unsupervised method to that of the *p* value.

## Supplementary Information

**Additional file 1.** This file includes the formal definitions related to Association Rules, the definition and computation of the Fisher's test and the EHR preprocessing used in this work.

## Data Availability

The data that support the findings of this study are available from Fuenlabrada University Hospital (HUF), but restrictions apply to the availability of these data, which were used under license for the current study, and so are not publicly available. Data are however available from the authors upon reasonable request and with permission from Fuenlabrada University Hospital.

## Abbreviations

AR: Association rule
PCDF: Polychlorinated dibenzofuran
ML: Machine learning
EHR: Electronic health record
ARM: Association rule mining
AC: Associative classification
DM: Data mining
HIV: Human immunodeficiency viruses
HUF: Fuenlabrada university hospital
ISO/EN: International Standards Organization European Norm
XML: Extensible mark-up language
ICU: Intensive care unit
PPM: Personalized and precision medicine

## Footnotes

1 http://www.philippe-fournier-viger.com/spmf/
