# Supplementary material for: Semi-supervised incremental learning with few examples for discovering medical association rules
Source: BMC Med Inform Decis Mak. 2022 Jan 24;22:20. doi: 10.1186/s12911-022-01755-3 (PMC8785547; doi:10.1186/s12911-022-01755-3)
Supplement: Supplementary file 1 — Additional file 1. This file includes the formal definitions related to Association Rules, the definition and computation of the Fisher's test and the EHR preprocessing used in this work. [file 12911_2022_1755_MOESM1_ESM.pdf]

Supplementary material of Semi-supervised Incremental Learning with Few Examples for Discovering Medical Association Rules

Ricardo Sánchez-de-Madariaga[a,*], Juan Martinez-Romo[b], José Miguel Cantero Escribano[c], Lourdes Araujo Serna[b]

[a] *Telemedicine and e-Health Research Unit, Instituto de Salud Carlos III, Monforte de Lemos 5, 28029 Madrid, Spain*
[b] *Languages and Information Systems Dpt., ETS Ingeniería Informática (UNED), Juan del Rosal 16, 28040 Madrid, Spain*
[c] *Preventive Medicine Service, Hospital Universitario La Paz-Carlos III-Cantoblanco, 28046 Madrid, Spain*

1. Formal Definitions related to Association Rules

A formal definition of an AR [1] is the following:

Association Rule (AR)

Let R be a set of binary attributes. Rule $X = x \rightarrow A = a$, where $|X| = l$, $x \in \{0, 1\}, l$ and $a \in \{0, 1\}$, is a dependency rule, if $P(X = x, A = a) \neq P(X = x)P(A = a)$. The dependency is positive, if $P(X = x, A = a) > P(X = x)P(A = a)$, and negative, if $P(X = x, A = a) < P(X = x)P(A = a)$.

Otherwise, the rule is called an independence rule.

In this paper we assume that all attributes in X are 1-valued and also a = 1, i.e. we consider rules with an only consequent.

We also give the formal definition of a statistical goodness measure, which is a function of data size n and frequencies P(XA), P(X) and P(A).

Goodness measure

Let $f : R^3 \times N \rightarrow R$ be a measure function, whose parameters are rule frequency $fr = P(XA)$, frequency of the condition part X, frX = P(X), frequency of the consequence A, frA = P(A), and the size of the data set n. If high values

*Corresponding author
Email address: ricardo.sanchez@isciii.es (Ricardo Sánchez-de-Madariaga)

of f (fr, frX, frA, n) indicate good dependency rules, then we say that f is increasing by goodness, and if low values of f (fr, frX, frA, n) indicate good rules, it is called decreasing by goodness. Different goodness measures are available. The most used are the χ^2-measure, for high absolute frequencies, and Fisher's exact test, when these frequencies are low in general.

2. Fisher test

Fisher test provides the significance of the association (contingency) between the two kinds of classification. The computation of the test is usually based on the contingency table recording the different classes. The p-value is computed as the hypergeometric distribution of the numbers contain in the cells of the table.

These two sets of rules allow us to apply the Fisher test to obtain the p-values for the rules in the holdout set. This is done by building for each rule R: $A \longrightarrow B$ in the holdout set a contingency table with the following data for the rule collected in the training set:

	rules with B (n_2)	rules without B ($N - n_2$)
rules with A (n_1)	rules with A and B (k)	rules with A (without B) ($n_1 - k$)
rules without A ($N - n_1$)	rules with B (without A) ($n_2 - k$)	rules without A and B ($N - n_1 - n_2 + k$)

The Fisher test applies a geometric distribution to the data in the table:

$$p(R) = \frac{\binom{n_1}{k}\binom{N-n_1}{n_2-k}}{\binom{N}{n_2}} \tag{1}$$

where N is the number of rules in holdout set, K is the number of rules in this set containing A and B, n_1 is the number of rules containing A, n_2 is the number of rules containing B.

3. Data Preprocessing

Narrative notes associated to EHR are written in natural language which gives them great variability when referring to the same medical condition. Besides, they often contain abbreviations and acronyms, and they also tend to contain spelling errors, and incorrect syntactic structures. Because of this, the normalization of clinical texts is paramount for extracting useful information [2]. We performed some simple preprocessing to our Spanish EHRs aimed at identifying the references to the same medical condition that is expressed in different ways. This process increases the number of cases for each condition, thus helping to obtain more reliable results.

The first transformation step of the normalization process is to convert the words to a canonical form, including all letters being lower case, removing punctuation, accent marks and other diacritics, reducing sequences of white spaces to one, and expanding some abbreviations.

The next step was to put medical conditions into their most simple form. For example, all the expressions like "unobjective respiratory distress", "Objective respiratory distress" and "severe respiratory distress" are changed to one heading, namely "respiratory distress".

Finally, we have separated some conditions mentioned in the same expression. For example, "cough and headache", has given rise to the two conditions "cough" and "headache".

After the normalization process, we keep only those medical conditions that have appeared more than once. In this way, we try to get rid of incorrect expressions that the normalization has not been able to transform into a recognizable case.

References

[1] W. Hämäläinen, Kingfisher: an efficient algorithm for searching for both positive and negative dependency rules with statistical significance measures, Knowl. Inf. Syst. 32 (2) (2012) 383–414.

[2] J. Pathak, K. Bailey, C. Beebe, S. Bethard, D. Carrell, P. Chen, D. Dligach, C. Endle, L. Hart, P. Haug, S. Huff, V. Kaggal, D. Li, H. Liu, K. Marchant, J. Masanz, T. Miller, T. Oniki, M. Palmer, K. Peterson, S. Rea, G. Savova, C. Stancl, S. Sohn, H. Solbrig, D. Suesse, C. Tao, D. Taylor, L. Westberg, S. Wu, N. Zhuo, C. Chute, Normalization and standardization of electronic health records for high-throughput phenotyping: The sharpn consortium, Journal of the American Medical Informatics Association : JAMIA 20 (E2) (2013). doi:10.1136/amiajnl-2013-001939.
